# Progress of Epidemics in Thirty-Eight Towns and Districts

**Published:** 1858-04

**Authors:** 


### PROGRESS OF EPIDEMICS.

### LOCAL REPORTS OF EPIDEMIC AND ENDEMIC DISEASES

*During the Months of December, January, and February,* 1857-8.

| Place. | County. | Lat. | Long. | Observer. |
|---|---|---|---|---|
| St. Mary, Scilly | Cornwall - | 49.50 N. | 6.24 W. | J. G. Moyle, Esq. |
| Teignmouth - | Devonshire - | 50.32 N. | 3.29 W. | W. C. Lake, Esq. |
| Odiham - - | Hampshire - | 51. 8 N. | 1. 3 W. | J. McIntyre, M.D. |
| Canterbury - | Kent - - | 51.17 N. | 1. 4 E. | G. Rigden, Esq. |
| Chatham - | Kent - - | 51.21 N. | 0.14 E. | F. J. Brown, M.D. |
| Wandsworth - | Surrey - - | 51.28 N. | 0. 7 W. | G. E. Nicholas, Esq. |
| Putney - - | Surrey - - | 51.28 N. | 0. 8 W. | R. H. Whiteman, Esq. |
| Up. Holloway | Middlesex - | 51.32 N. | 0.03 E. | W. B. Kesteven, Esq. |
| Swansea - | Glamorgansh. | 51.38 N. | 3.50 W. | W. H. Michael, Esq. |
| Colchester - | Essex - - | 51.54 N. | 0.52 E. | Henry Laver, Esq. |
| Aspley Guise - | Bedfordshire - | 52. 1 N. | 0.37 W. | J. Williams, M.D. |
| Saffron Walden | Essex - - | 52. 3 N. | 0.12 E. | T. Spurgin, Esq.<br>H. Stear, Esq. |
| Bedford - - | Bedfordshire - | 52. 8 N. | 0.51 W. | T. H. Barker, M.D. |
| Newpt. Pagnell | Buckinghams. | 52.10 N. | 0.42 W. | G. O. Rogers, Esq. |
| Sharnbrook - | Bedfordshire - | 52.12 N. | 0.40 W. | R. S. Stedman, Esq. |
| Wellingbro' - | Northamptons. | 52.20 N. | 0.40 W. | B. Dulley, Esq. |
| Beccles - - | Suffolk - - | 52.25 N. | 1.48 E. | W. E. Crowfoot, Esq. |
| Thetford - | Norfolk - - | 52.26 N. | 0.45 E. | H. W. Bailey, Esq. |
| Stourbridge - | Worcestersh. - | 52.28 N. | 2.32 W. | A. Freer, Esq. |
| Dudley - - | Staffordshire - | 52.30 N. | 2.10 W. | J. H. Houghton, Esq. |
| Barrowden - | Rutland - | 52.34 N. | 0.38 W. | H. J. Swann, Esq. |
| Wisbeach - | Cambridgesh. | 52.39 N. | 0. 5 E. | W. H. Hole, Esq. |
| East Dereham | Norfolk - - | 52.40 N. | 0.57 E. | J. Vincent, M.D. |
| Pontesbury - | Shropshire - | 52.43 N. | 2.50 W. | Wm. Eddowes, Esq. |
| Nottingham - | Nottinghamsh. | 52.50 N. | 1.10 W. | T. Robertson, M.D. |
| Burton-on-Trt. | Staffordshire - | 52.53 N. | 1.53 W. | S. Thomson, M.D. |
| Oswestry - | Shropshire - | 52.53 N. | 2.59 W. | P. Cartwright, Esq. |
| Wrexham - | Denbighshire - | 53. 2 N. | 3. 1 W. | E. Williams, M.D. |
| Hawarden - | Flintshire - | 53.11 N. | 3. 2 W. | T. Moffat, M.D. |
| Lincoln - | Lincolnshire - | 53.12 N. | 0. 5 W. | S. Lowe, Esq. |
| Alford - - | Lincolnshire - | 53.15 N. | 0. 6 E. | R. U. West, M.D. |
| Gainsborough | Lincolnshire - | 53.23 N. | 0.47 W. | D. Mackinder, M.D. |
| Liverpool - | Lancashire - | 53.24 N. | 2.59 W. | Thos. Bickerton, Esq. |
| Warrington - | Lancashire - | 53.25 N. | 2.32 W. | C. N. Spinks, Esq. |
| Wigan - - | Lancashire - | 53.32 N. | 2.33 W. | Jephtha Hussey, Esq. |
| Bolton - - | Lancashire - | 53.35 N. | 2.19 W. | W. H. Pendlebury, Esq. |
| Bramley - | Yorkshire - | 53.49 N. | 1.38 W. | J. N. Radcliffe, Esq. |
| York - - | Yorkshire - | 53.58 N. | 1. 3 W. | W. Procter, Esq. |
| Wst. Auckland | Durham - | 54.45 N. | 1.40 W. | G. Todd, Esq. |
| Rothbury - | Northumberld. | 55.25 N. | 1.50 W. | E. C. Summers, Esq. |
| Lerwick - | Shetland Isles | 60. 9 N. | 1. 8 W. | G. W. Spence, M.D. |

### QUARTERLY STATEMENT—No. XIII.

[*The dates denote that the disease appeared in the weeks then ending.*]

SCARLET FEVER.

Canterbury—January 15-29, Feb. 5
Wandsworth—Jan. 15, Feb. 5-12, 26
Putney—Every week
Upper Holloway—Jan. 15, Feb. 5
Colchester—February 12-19
Beccles—Every week
Thetford—December 4
Stourbridge—January 1, 22-29
Dudley—January 15-29
Wisbeach—December 4, 11
Nottingham—All Dec., Jan. 8

Oswestry—February 5-19
Wrexham—December 4-18
Hawarden—January 22, February 26
Lincoln—Dec. 18-25, Jan. 8-29, Feb. [5-12
Gainsborough—Every week
Liverpool—Every week
Warrington—December 18
Hindley, Wigan—January 8, 22
Bolton-le-Moors—Dec. 4-18, Jan. 8-29, [all Feb.
Bramley—Dec. 11, Jan. 22-29
York—Dec. 4-18, Jan. 1-8, 22-29
West Auckland—February 12-26

MEASLES.

Odiham—February 26
Canterbury—Jan. 22-29, all Feb.
Chatham—Dec. 11, Jan. 8, all Feb.
Saffron Walden—February 26
Beccles—Every week
Stourbridge—February 12-19
Dudley—February 12-19
Barrowden—January 8-15
Wisbeach—February 5-12
Nottingham—Dec. 18, Jan. 29
Oswestry—December 25, January 1
Wrexham—February 26
Hawarden—December 18, January 1
Liverpool—Every week
Bolton-le-Moors—Dec. 11-25, Jan. 15-22, all February

SMALL-POX.

Odiham—February 26
Bedford—Dec. 25, all Jan. and Feb.
Wellingborough—December 4-18
Stourbridge—January 1
Wisbeach—Every week
Pontesbury—Every week
Burton-on-Trent—February 26
Oswestry—February 26
Wrexham—All December, Jan. 1-22
Gainsborough—All Jan., Feb. 5-19
Liverpool—Every week
Warrington—Dec. 18, Jan. 8, 29, Feb. 5
Hindley, Wigan—Dec. 11, 25, Jan. 8, 22, Feb. 5-12, 26
Bolton-le Moors—Dec. 18-25, Jan. 1, [22-29
York—All Dec. and Jan., Feb. 12

HOOPING COUGH.

Canterbury—Every week
Chatham—Dec. 4-11, Jan. 22, Feb. 19
Upper Holloway—Dec. 18-25, Jan. 22-[29, all Feb.
Colchester—Dec. 4-11
Saffron Walden—Every week
Newport Pagnell—Jan. 15-29, all Feb.
Sharnbrook—Dec. 11-25, Jan. 1-15
Wellingborough—December 11-25
Stourbridge—February 5
Dudley—January 29, February 19
Wisbeach—Every week
Nottingham—February 5
Oswestry—Every week
Alford—January 1, February 12-26
Gainsborough—All Jan. and Feb.
Liverpool—Every week
Warrington—January 15
Hindley, Wigan—Dec. 11, 25, Jan. 15, Feb. 5
Bolton-le-Moors—Dec. 4-18, Jan. 8-22, [Feb. 5, 19-26

CROUP.

Teignmouth—December 18
Odiham—All February
Canterbury—December 25, Jan. 29
Chatham—February 5, 26
Upper Holloway—Dec. 11, Jan. 22
Aspley Guise—December 4-11
Saffron Walden—December 4-11
Newport Pagnell—Dec. 4, Jan. 29
Sharnbrook—February 12
Beccles—February 19-26
Thetford—December 11
Stourbridge—December 4, Jan. 15
Barrowden—February 5
Pontesbury—February 19
Burton-on-Trent—Jan. 15, Feb. 12
Nottingham—January 8
Oswestry—January 1, February 12
Wrexham—January 29, February 5-19
Lincoln—Dec. 18-25, Jan. 1, 22-29, [Feb. 5
Alford—February 12
Liverpool—Dec. 18-25, all Jan. & Feb.
Hindley, Wigan—February 5-19
Bolton-le-Moors—Dec. 11, 25, Jan. 1, 29, February 5

CATARRH.

Teignmouth—Dec. 4, 18-25, all Jan. & [Feb.
Odiham—Every week
Chatham—Dec. 4-12, Jan. 1, 22-29, all [Feb.
Wandsworth—Every week
Putney—All December and January
Upper Holloway—Dec. 18-25, all Jan., [Feb. 5-19
Colchester—Every week
Aspley Guise—January 15
Saffron Walden—Every week
Bedford—January 29, February 5-12
Newport Pagnell—December 4-11, all January and February
Sharnbrook—All Dec. & Jan., Feb. 5-19
Wellingborough—Every week
Beccles—Dec. 18-25, Jan. 1, Feb. 5-12
Thetford—Every week
Stourbridge—Every week
Dudley—December 18, January 8, 29
Wisbeach—Every week
Nottingham—Dec. 25, all January
Burton-on-Trent—Every week
Oswestry—Every week
Alford—February 5
Gainsborough—Every week
Warrington—Dec. 4-11, 25, Jan. 8, 22, [Feb. 5
Bolton-le-Moors—Dec. 11-25, Jan. 1, 22-29, February 5-12

Bramley—January 29, February 5-12
York—Dec. 11-18, Jan. 1-15, 29, Feb.
Lerwick—Dec. 4-18, all Jan. [5-19

INFLUENZA.

Teignmouth—Dec. 4-18, Jan. 8, Feb. 5
Odiham—December 4-11 [Feb.
Chatham—Dec. 11-25, Jan. 1, 22 29, all
Wandsworth—Dec. 4-18, Jan. 8-22
Putney—All Dec., Jan. 1, 22-29
Upper Holloway—January 8
Wanstead—All Dec. & Jan., Feb. 5-12
Colchester—Every week
Aspley Guise—Dec. 11-25, Jan. 8
Saffron Walden—Every week
Bedford—Dec. 25, Jan. 8-15, 29
Newport Pagnell—Every week
Sharnbrook—All Dec. & Jan., Feb. 12-
Wellingborough—Every week [19
Beccles—January 8-22
Thetford—Every week
Stourbridge—All Dec. and Jan.
Dudley—December 11
Barrowden—All January and Feb.
Pontesbury—Dec. 25, Jan. 1, all Feb.
Nottingham—Jan. 8 29, Feb. 5-12
Wrexham—Jan. 1-22
Lincoln—Dec. 11-25, Jan. 1, 15-29, Feb.
Liverpool—Every week [5-19
Warrington—Dec. 4, 18, Jan. 29, Feb. 26
Hindley, Wigan—Jan. 15-22
Bolton-le-Moors—All Dec., Jan. 1-22,
Bramley—Jan. 15-22 [Feb. 5-12
York—All Dec., Jan. 1-8, 22-29, Feb. 5
Lerwick—Dec. 11-25, all Jan., Feb. 5, 19

ERYSIPELAS.

Teignmouth—Dec. 25, Jan. 15, Feb. 12
Canterbury—Dec. 25, Jan. 8, 29
Chatham—Dec. 4, 18-25, Jan. 1, 29, February 5, 19
Wandsworth—Jan. 1, 15, Feb. 12, 26
Putney—February 5
Upper Holloway—February 5 [12-19
Colchester—Dec. 18-25, Jan. 15, Feb.
Saffron Walden—Jan. 22, Feb. 19
Bedford—February 26
Newport Pagnell—December 11-18
Sharnbrook—Jan. 29, Feb. 5, 19
Wellingborough—January 15-29
Beccles—December 11
Thetford—December 4, 25, Feb. 12
Stourbridge—Dec. 25, Jan. 15, Feb. 5
Dudley—January 15
Barrowden—February 12
Wisbeach—December 4-18
Pontesbury—February 19-26 [5-19
Nottingham—Dec. 18, Jan. 22-29, Feb.
Oswestry—February 19-26
Alford—December 4, 18, January 22
Gainsborough—February 26
Liverpool—Every week
Hindley, Wigan—January 15-22
Bolton-le-Moors—Dec. 4-18, Jan. 15-
Bramley—Feb. 19 [29, Feb. 5-12
York—February 12
Lerwick—December 4, January 15, 29

AGUE.

Canterbury—Every week
Chatham—Dec. 6-18, all Jan., Feb. 12-
Colchester—Every week [26
Bedford—All February
Thetford—Dec. 18, Jan. 8, Feb. 12-26
Barrowden—February 12
Wisbeach—All January and February
Wrexham—Dec. 18-25, January 1

REMITTENT FEVER.

Teignmouth—December 18, Jan. 8
Odiham—February 19-26
Upper Holloway—January 8
Saffron Walden—All Dec., Jan. 1-22
Newport Pagnell—December 18
Thetford—Dec. 4-11, Jan. 1, 22, Feb. 19
Stourbridge—Jan. 15-29, Feb. 12, 26
Oswestry—Every week
Wrexham—January 15-29, February 5
Hawarden—January 22
Alford—Every week
Warrington—Dec. 4, 25, Jan. 22-29
Hindley, Wigan—Jan. 22, Feb. 5
Bolton-le-Moors—Dec. 18-25, Jan. 1-8, 29, all February

DIARRHŒA.

Teignmouth—Dec. 4, 18-25, Jan. 1-8, 22, February 12-26
Canterbury—Dec. 25, Jan. 8-29, all Feb.
Chatham—Dec. 4, 18-25, Jan. 1-8, 22-29, Feb. 5, 19-26 [all Feb.
Wandsworth—Dec. 4, 18-25, Jan. 1-22,
Putney—Dec. 18 25, Jan. 8-22, Feb. 12
Upper Holloway—Jan. 22-29, Feb. 5-12
Aspley Guise—Dec. 4-11, 25, Jan. 15-22,
Saffron Walden—All Dec. [Feb. 12-19
Bedford—All February
Newport Pagnell—Jan. 8-29, Feb. 5
Sharnbrook—All Dec., Jan. 29, Feb. 12
Beccles—January 8-22
Thetford—Dec. 4-11, 25, Jan. 1-8, 29, all
Stourbridge—Dec. 25, Jan. 1 [Feb.
Dudley—All Dec. and Jan., Feb. 12-19
Barrowden—Dec. 18-25, Jan. 1-8, 22, February 5-19
Wisbeach—All January and February
Nottingham—Dec. 4-18, Feb. 19
Burton-on-Trent—Dec. 11, Jan. 1, 15
Oswestry—All January and February
Hawarden—Dec. 11-18, Jan. 8-29, Feb.
Alford—January 1, all Feb. [5-10
Gainsborough—Dec. 18-25, all Jan. &
Liverpool—Every week [Feb.
Bolton-le-Moors—Dec. 4-18
York—Dec. 11, 25, Jan. 15-22, Feb. 12

Oswestry—January 1
Wrexham—February 19-26
Lincoln—January 29, February 5-12
Alford—December 25, February 12-26
Gainsborough—Dec. 18-25, all Jan. & [Feb.
Liverpool—Every week
Warrington—February 5
Hindley, Wigan—Jan. 8-22
Bolton-le-Moors—Dec. 18-25, Jan. 1, 22-29
Bramley—Dec. 25, Jan. 1, 15-22
York—December 4, 25, January 1
Lerwick—Dec. 11, Jan. 15-22, Feb. 5, 19

TYPHUS.

Teignmouth—Dec. 25, Jan. 22, Feb. 12
Odiham—February 12
Canterbury—Every week
Chatham—Dec. 4-18, Jan. 8-15, 29, February 12
Wandsworth—Dec. 11, Feb. 5, 19-26
Putney—January 15-22
Saffron Walden—All December
Bedford—Dec. 11-18, Jan. 1, 15-29, Feb. [5
Newport Pagnell—Every week
Wellingborough—All Dec. and Jan.
Beccles—Dec. 4-18, Jan. 29, Feb. 5-12
Thetford—All December, January 8
Stourbridge—January 1
Dudley—Every week
Wisbeach—January 8-29, February 5
Pontesbury—Every week
Lerwick—Dec. 11-18, Jan. 1, 22-29, February 12

DYSENTERY.

Odiham—February 19-26
Chatham—Dec. 4-11, all Jan. Feb. 12-19
Wandsworth—Jan. 1, 29, Feb. 19-26
Newport Pagnell—All February
Stourbridge—December 11-18
Nottingham—Dec. 4, 18-26, Jan. 1-22, [Feb. 5
Oswestry—February 5-12
Alford—All December and January
Gainsborough—All Jan. and Feb.
Bolton-le-Moors—All Dec., Jan. 1-15, 29, February 5, 19-26
West Auckland—All December

TYPHOID FEVER.

Colchester—Every week

PUERPERAL FEVER.

Chatham—January 15, February 5
Beccles—All February
Thetford—December 11

CARBUNCLE.

Teignmouth—Dec. 4, January 8
Chatham—January 22-29
Upper Holloway—February 26
Colchester—All February
Saffron Walden—Dec. 4, January 8
Bedford—December 18
Sharnbrook—Jan. 15, 29, Feb. 5-12
Wellingborough—Jan. 8-29, all Feb.
Beccles—January 8-22
Stourbridge—December 4
Nottingham—Dec. 25, Feb. 5.
Burton-on-Trent—December 11
Lincoln—February 12-26
Bolton-le-Moors—Dec. 25, Jan. 1, Feb. [5-19
York—Dec. 18, Jan. 8, 22-29, Feb. 5

CHICKEN-POX.

Wandsworth—February 26

MUMPS.

Canterbury—February 19
Lerwick—Dec. 4, February 19-26

DIPHTHERITE.

Canterbury—All January and Feb.
Colchester—Jan. 22-29, Feb. 19-26

APHTHOUS ULCERATION OF MOUTH.

Lerwick—December 11, January 15

---

### ADDITIONAL OBSERVATIONS.

*St. Mary's, Scilly.*—Mr. Moyle returns a clean bill of health, with the following remarks:—

"The quarter has been very healthy. We have had no epidemics. The ozone average per week has been 3. The weather has been delightful; only one snow-storm for the winter."

*Teignmouth.*—Mr. Lake writes: "The high rate of temperature observed during the last two quarters continued through December, the temperature for this month being 48·8°. A sudden fall of temperature took place in the early part of January, the lowest minimum point for the quarter (23·8°) being registered on the 6th of this month. Through January and

February the temperature was somewhat variable, though, for the most part, mild; but between the mean temperature of December and that of January there was a difference of 6·8°; between that of January and of February, a difference of 1·8°. February closed with cold and bleak weather, and on the last two days of the month snow fell. The barometric pressure was persistently very high through December and January; the mean pressure for the former month being 30·264 ins.; for the latter, 30·286. Between December 5th and January 26th, the mercury on two days only fell between 30·000. The mean pressure for February was 29·877. The total rainfall for the three months of the quarter was respectively 0·768 ins., 0·493, 1·275; the mean ozone, 6·0, 4·9, 5·8. The prevailing winds, during December, were from south and southwest; during January, from north, south-west, and south; during February, from north, north-east, and east. I have nothing particular to report about the diseases of the quarter. I have not seen nor heard of the occurrence of any more cases of diphtherite; nor did many occur at first. The number of deaths registered in the town, from all diseases, was unusually high during December."

*Odiham.*—The case of measles was imported from Worthing; the case of small-pox was brought from Aldershott camp. Diphtherite has prevailed epidemically, of a low type, attacking adults as well as children. Nitrate of silver, locally applied, quinine, stimulants, and nourishment, constituted the treatment. Several deaths occurred. The cases of croup occurred during an intensely cold wind.

*Canterbury.*—Mr. Rigden says: "During the past three months the following deaths have been reported in this city:

| | *Dec.* | *Jan.* | *Feb.* | *Total.* |
|---|---|---|---|---|
| Fever | 7 | 3 | 6 | 16 |
| Hooping cough | 1 | 7 | 4 | 12 |
| Erysipelas | 0 | 1 | 0 | 1 |
| Measles | 0 | 0 | 1 | 1 |
| Diarrhœa and catarrh | 1 | 0 | 0 | 1 |
| Croup | 0 | 1 | 0 | 1 |
| Diphtheria | 1 | 3 | 2 | 6 |

"There is, unfortunately, almost always fever of a low type prevalent in this city; but it is mostly confined to the poorest, overcrowded, and badly drained districts. Diphtheria has been prevalent in this city since July last; but, in my experience, it has not assumed so malignant a character during the last three months as it did during the earlier period of its progress."

*Chatham.*—Dr. Brown writes: "Neuralgia and ague have continued prevalent; and dysentery (which is unusual in winter) has affected a great many individuals, both young and middle aged. Metria occurred in two weeks, preceded by an unusual number of cases of abortion, some of which were attended by symptoms denoting a septic state of the system. I predicted the appearance of metria when I noticed these cases. The type of metria was paludal, with local congestion. Only a few cases occurred, and none proved fatal. There were also cases of phlegmasia dolens and inflammation of the breasts. In December a great number of children (many of them infants) suffered from inflammation of the kidneys, followed by anasarca, convulsions, and death. These cases were well marked; but renal disorder is common in infants, although not generally recognised. I have clearly ascertained that the urine is diminished in quantity, and sometimes suppressed for twenty-four to thirty-six hours before the occurrence of infantile convulsions. Probably uræmia is the invariable concomitant of convulsions. Nephritis in infants is detected by screaming just as if a pin had pricked the abdomen in its lateral aspect, accompanied by diminution of the renal secretion, and sometimes by vomiting."

*Wandsworth.*—Mr. Nicholas says: "The most prevalent and severe forms of disease during the past quarter were those of the respiratory organs, which, in three hundred and fifty-two pauper patients coming under my care, constituted upwards of one-third of the whole. With the exception of a few isolated cases of scarlatina of mild form, there has been an absence of the exanthemata; and no death has resulted from disease of the zymotic class during the period. The total mortality has been considerably below the average."

*Putney.*—Mr. Whiteman writes: "Scarlatina was frequent and severe throughout the entire quarter, but proved fatal in very few instances. Catarrh and influenza prevailed with considerable severity during December and January. Diarrhœa was of much more frequent occurrence during the latter part of December and the following month, than is generally observed at that season. The two cases of typhus that came under my notice were clearly traceable to bad ventilation and imperfect drainage. One case, when the removal of the patient was impracticable, proved very severe. The other was comparatively mild, the sufferer having been sent to the Fever Hospital early. A case of diphtherite came under treatment at the latter part of February, and proved fatal in five days from the first appearance of the disease."

*Colchester.*—During the last quarter, this district has suffered but little from disease. Typhoid fever cases are now rare.

Tonsillitis still continues prevalent; and there have been some few cases of diphtherite. Pleuropneumonia still prevails amongst cattle, but not to any great extent. The wind has been principally north-east and east; the weather dry.

*Saffron Walden.*—Mr. Spurgin writes: "Vegetation was unusually active till the commencement of January, when it received a check by slight frosts, the season being unusually dry. In February the weather became more severe, and continued so till the end; the prevalent winds being easterly and north-east, with very little snow, but much rain on the 4th. Small-pox has not been seen by me in this quarter."

Mr. Stear says that "the past quarter has been remarkably healthy in this neighbourhood, probably on account of the mildness and dryness of the winter."

*Bedford.*—Dr. Barker writes: "Small pox has prevailed during this quarter. The first case was clearly imported by a tramp. It has assumed a modified form generally, although severer, and even fatal cases, have occurred in a few instances. There are but few unvaccinated persons in this district. Fever of a typhoid character has prevailed; and a few cases of ague have occurred. Pleuropneumonia has occurred amongst cattle."

*Sharnbrook.*—Mr. Stedman writes: "Influenza has been the prevailing malady of the quarter. In many cases it has been complicated by bronchitis or pneumonia; but few fatal cases have occurred. The weather has been remarkably dry; and the barometer has stood at an unusual height all through the quarter."

*Beccles.*—Mr. Crowfoot states that measles and scarlet fever have been very prevalent during the last three months. The type of the latter disease has been very severe, several cases terminating fatally previously to the appearance of the eruption, and many being accompanied with severe diphtherite.

*Thetford.*—Mr. Bailey furnishes the following summary of the meteorology and diseases of the quarter:—

"December. The temperature of the month was very mild; and although the average difference between this and last December was only half a degree, yet, from the deficiency of wind, and the very little amount of rain, the weather was unusually fine. The range during the month was 33°. The barometrical diagram assumed that of a summer month, the deviation being but very little, and the prevalence of winds being from the south-west. Although the weather was so remarkably mild, yet the diseases of the month were numerous. Catarrh and influenza were most prevalent, scarcely a family escaping them. With children, bronchitic affections and pleuritic diseases were fatal in four cases. I have seldom known the disease so general in this month for some years. One case of spasmodic croup came under treatment; during the paroxysm it caused the greatest alarm, but the patient recovered in a few days. Quinsy, and both chronic and acute rheumatism, were also very general, occurring, more particularly, in the working classes ; as were also ague and remittent fever, four cases of the latter coming under treatment. Diarrhœa also prevailed throughout the month, attacking all grades, and leaving great prostration of strength. Typhus fever occurred in every week of this month, attacking the labourers, and running a long course. No immediate cause could be assigned, other than atmospheric influence. Special care was given to free ventilation, and the avoidance of nuisances in the locality. One case of puerperal fever took place, in a single woman, a few days after confinement ; it quickly terminated fatally. The casual diseases of the month under observation, were convulsions, consumption, apoplexy, abscess, paralysis, hysteria, hæmoptysis, fistula, menorrhagia, abortion, psora, porrigo, eczema, and acne. Amongst cattle, the lung disease was, in this locality, very prevalent and fatal. Homœopathy is cried up much by farmers, who believe that a globule placed upon the tongue of a bullock will do wonders : but the deaths amongst cattle were numerous. Catarrhal affections and glanders were also prevalent ; and two cases of inflammation of the bowels terminated fatally. From the mildness of the month it has been recorded, that currant-bushes budded almost into leaf, and also that raspberries were gathered. The wheat assumed a most luxuriant aspect. The quantity of rain during the year has only been fourteen inches, being less than the average by eight inches.

"January 1858. Since January 1855, I have not recorded so little fluctuation of the barometer as in this month, and probably to the same cause may be attributed the little wind and rain, the latter amounting to little more than a quarter of an inch, and only five days of wind. The glass ranged between 30· and 30·60. It was not so in the thermometical range, there being great changes of temperature during the day. Some nights were very severe. On the 6th the thermometer was as low as 18°, and the next day as high as 50°. These vicissitudes of temperature occasioned the prevalence of influenza, which was unusually epidemic, scarcely a family escaping its influence. It was fatal in one case, occurring in a feeble elderly person. With children it was frightfully prevalent, and to the very young, or infants, it was fatal. Quinsy and common sore throats were also general; the former more particularly so than for many years. Only one case of ague, and one of remittent fever took place; and diarrhœa was less frequent than in December; and only one case of typhus occurred. The casual diseases of the month, and under medical notice, were syphilis, hæmorrhoids, dyspepsia, enlargement of the liver with dropsical effusion, nervous diseases, and some cutaneous complaints. The same diseases existed in cattle as in last month, now attacking cows during calving, and ending fatally. Among sheep, no diseases were recorded.

"February. Although in the former months of February we experienced some few days of low temperature and nights of severe frosts, yet, taking the average of the month, altogether it was colder. The highest temperature was 48°, the lowest 20°, and on the grass 18°. Thus the temperature assumed a more wintry range than usual. The barometical range was, throughout the month, less fluctuating. The prevailing diseases were catarrhal affections and influenza; diarrhœa, which was more general; sore throats; ague; and there was a case of remittent fever. Some cases occurred, in children, of bronchitis, from exposure to the easterly winds; and two cases of diphtherite, of a mild character, came under notice, but this disease has not assumed an epidemic nature. Several cases of consumption terminated this month; and also four cases of inflammation of the lungs. The casual diseases of the month were phlegmonous inflammation of the leg, gout, rheumatism, enlargement of the heart, chronic disease of the liver, epistaxis, hydrocele, atrophy, spinal disease, and convulsions. With cattle there seemed less disease, but it was of the same character as in last month. Cows in calving did badly, and several died. Sheep were healthy. Vegetation has fortunately been kept back by the frosts."

***Record of Deaths in Twenty-one Parishes** (population 9,574) **from the Registrar's Book.***

| *December* 1857. | | *January* 1858. | | *February.* | |
|---|---|---|---|---|---|
| Diarrhœa ........ | 1 | Influenza ........ | 1 | Consumption ...... | 7 |
| Puerperal fever .... | 1 | Consumption ...... | 1 | Inflamm. of lungs .. | 4 |
| Consumption ...... | 2 | Croup ............ | 1 | Congestion of lungs | 3 |
| Bronchitis ........ | 4 | Apoplexy.......... | 1 | Croup ............ | 1 |
| Pleuritis .......... | 1 | Congestion of brain. | 1 | Jaundice .......... | 1 |
| Apoplexy.......... | 1 | Atrophy .......... | 1 | Congestion of brain | 1 |
| Convulsions ...... | 1 | Debility .......... | 1 | Spinal disease...... | 1 |
| Paralysis.......... | 1 | Decay of nature .... | 1 | Convulsions ...... | 2 |
| Abscess .......... | 1 | | | Dropsy............ | 1 |
| Debility .......... | 5 | | | Debility .......... | 3 |
| Decay of nature .... | 1 | | | Decay of nature .... | 5 |
| | 19 | | 8 | | 29 |

*Stourbridge.*—Bronchitis prevailed among children during the last fortnight of the quarter. In many houses three, or four, or five, were affected. It was very fatal in infants only a few weeks old, about the third, fourth, fifth, or sixth day of the illness. The winds were chiefly from the east.

*Dudley.*—Mr. Houghton says: "Though my report does not shew the existence of small pox in Dudley, the registration of seven deaths from that cause proves that it still exists, though only very slightly as compared with former reports. Deaths from scarlet fever, hooping cough, and croup, are on the increase. Were some of the cases reported as croup cases of diphtherite? I think this probable. Two deaths are registered from diphtherite; and one case (not fatal) of the disease has fallen under my care in the last quarter. It was attended with profound prostration, and yielded, as other cases had done, to nutrients and stimulants freely used, and tincture of sesquichloride of iron with hydrochloric acid. The deaths from diarrhœa and fever were few for Dudley; thirty-six deaths were caused by convulsions, or one in eleven of the whole deaths. The number of deaths registered were 397; the average of nine corresponding quarters is 302: the excess being 95.

"The drainage of the town will be commenced this spring, and it is hoped that the excessive mortality so long and notoriously existing, will be diminished.

"A grave typographical error occurred in my last report from Dudley (vol. iii, p. 408, l. 30). It is there made to appear, that my cases of diphtherite occurred under the 'best' sanitary conditions; whereas the very reverse was the fact. Two of the cases occurred in the immediate vicinity of slaughter-houses, where *pigs* also were kept; and in one of these two places complaint had been made of a foul sewer. Almost immediately after my patient recovered from the attack of diphtherite, and whilst she was yet in the country for change of air, her husband was seized with typhus of a bad character, from which he escaped with difficulty. The third case happened in a low part of the town, and was in close relation to some foul *privies and pigsties.* In a subsequent case, the disease was associated with an offensive open drain, running along the gable end of the house, and close to the sittingroom and bedroom which the patient occupied."

*Barrowden.*—Mr. Swann writes: "The weather up to February was unusually mild, dull, and dry. The wind was south-west the whole winter; in the beginning of February it veered round eastward, since which time we have had it cold, with some rain. Influenza has been unusually severe; it has not before been so prevalent in this neighbourhood for many years.

At Haringworth, measles have been very severe all the quarter; a few cases have proved fatal. Diarrhœa, too, has remained with us, but has not been so severe as in last quarter."

*Wisbeach.*—Mr. Hole says that "so long a drought, at this season of the year, has scarcely been known in the memory of the 'oldest inhabitant.' The drains and dikes have been nearly all empty. People are drinking water not fit for cattle; and much diarrhœa and sickness is the result."

*Pontesbury.*—Mr. Eddowes says: "Small-pox has continued here during the quarter; the cases have mostly been isolated. There were three cases only in one parish, containing a population of upwards of 900 inhabitants. Two of them had had small-pox previously; the third had been vaccinated about twenty years ago: he had the disease very mildly, only having about half a dozen pocks on his face. Of the two who had had small-pox previously, the one was twenty-one years of age; she had had small-pox when an infant a few weeks old: the other was a woman upwards of fifty years of age; she had been inoculated, and had the disease just before vaccination became general. The latter two had the disease very severely. The young woman, who was twenty-one years of age, got through with the greatest difficulty."

*Burton-on-Trent.*—The epidemic and endemic diseases of the last quarter have been little varied. Affections of the respiratory organs, and occasional diarrhœa, have been the most prevalent. In the town of Burton-on-Trent, small-pox has been very extensively diffused, and many deaths have occurred.

*Gainsborough.*—Dr. Mackinder writes: "Since my last report, this neighbourhood has been, and is still, suffering from epidemic throat affection in all its varieties, save that which has a fatal termination. Scores of cases have been under my own observation, and doubtless many have been attended by other practitioners, and all have required the tonic treatment. As previously described, in the majority of cases, the local manifestation of this general disease has been a congested rubeolated condition of the fauces, uvula, and palate, sometimes accompanied by inflammation of the tonsils and mouth, (simple and ulcerative), and of the tongue, while, far from unfrequent, are fully developed specimens of diphtheria, with bronchitis, gastro-enteritis, diarrhœa, dysentery, and fever. Pain is still the exception in these cases. Lassitude is much complained of; prostration is occasionally present. Sometimes there is a dry incessant cough, which, though occasionally relieved by the application of nitrate of silver, often sets all treatment at defiance. The parotid glands are frequently involved; and a rash, like that of scarlatina, is sometimes seen on the face and chest; in one case I saw it on the hands, which were swollen. Another patient had a little eruption on the chest, which came out on three successive evenings. She was confined to her bed for a few days; and after her recovery, her infant was seized with scarlatina, the rest of the family escaping. Surely diphtherite must be closely allied to, or identical with, scarlatina, the *materies morbi* being meted out in less effective doses? There is a disposition to recur, in some of the fully developed cases of diphtherite; second and third crops of minute shot-like deposits of lymph having been observed. Many of the worst cases under my care have been the most amenable to treatment; the milder ones assuming a chronic and persistent form, without much detriment to the general health. One of my cases, which appeared mild at first, ran into typhoid, in consequence of exposure to the effluvium of an untrapped sewer. Another had a severe attack of erysipelas of the face and head. In addition to the chlorate of potassa, I have given hydrochloric acid, with preparations of iron and quinine; and, in some cases, continue to apply the solid nitrate of silver with advantage; the diet being of the most liberal description. A woman under Mr. Sharp, at the dispensary, required a bottle of wine daily for some time: she recovered.

"We have had but a few cases of (fully developed) scarlatina; all did well. Small-pox was introduced from Sheffield on the first of January—an enviable new year's gift! The first case was a child one year old. The eruption appeared four days after its arrival here. While at Sheffield, it used to run into the house of a neighbour affected with small-pox. The next was an adult from the same town, and from him the third took it by the intercommunication of another party. One woman had the small-pox a few weeks before her accouchement, and the infant was born with a few pustules, which afterwards maturated. Altogether I have heard of nine cases, all of which I believe had been vaccinated; and no fresh ones have been reported for several weeks.

"Erysipelas, rheumatism, gout, colic, and pneumonia, have occasionally occurred: diarrhœa, dysentery, bronchitis, and hooping cough, have made a longer stay among us; while fever has been performing the part of the shy and diffident stranger. I have seen one case of sudden death from valvular disease of the heart; one, in an infant, from smothering; and one from the plugging up of a large artery. The subject of this sudden arrest of the circulation, was the wife of a respectable tradesman; she had fifteen days previously given birth to her second child. The labour was natural, easy, and of short duration, and the only thing requiring attention afterwards was an excessive secretion of milk. On February 21st she sat up to her tea and supper, appearing well, and in good spirits, though she complained of being fatigued when she returned to bed. At four on the following morning she gave the child the breast, commenting playfully on the abundant supply of milk; and a few minutes before five she rose up convulsively, said she was choking, and breathed her last. In consequence of the suddenness of the death, the cause of which was suspected, I was allowed to make a *post mortem* examination, and found a large branching fibrinous plug completely stopping up the right pulmonary artery, and its immediate ramifications; while the entrance to the left pulmonary artery gave lodgment to a large and tolerably firm concretion. The heart was rather thin, and the lungs were slightly congested; but there was no further trace of disease about the body. One woman, in whom the operation of transfusion of blood had been previously performed, died from anasarca. At Morton, a village about a mile distant, a boy, aged twelve, had inflammation of the fauces, followed by bronchitis, hæmatemesis, exudation of blood from the fauces, vesicles of pemphigus on both knees, and typhoid with delirium. He recovered, after an abundant supply of wine, beef tea, quinine, and iron."

"Herewith I send a daily register of cases that have been under my care for the past three months. I have not given the number of patients, but merely the names of the diseases, as recorded in my books, when treatment commenced. My next will embrace the daily observations on meteorology, by Mr. Dyson, so arranged as to show the connexion between disease and the weather."

| | *Diseases of Private Patients.* | *Diseases of Pauper Patients.* |
|---|---|---|
| | 1857. December. | |
| 1. | Faucitis,* catarrh, rheumatism, [diarrhœa. | Dysentery, tonsillitis. |
| 2. | Dysentery. | Catarrh, bronchitis. |
| 3. | Faucitis, scarlatina, diarrhœa. | Menorrhagia. |
| 4. | | Catarrh, diarrhœa, bronchitis. |
| 5. | Dysentery, bronchitis, faucitis. | Typhoid, bronchitis. |
| 6. | | Bronchitis. |
| 7. | | Dyspepsia, fever, diarrhœa. |
| 8. | Faucitis. | Bronchitis. |
| 9. | Bronchitis. | Hæmatemesis. |
| 10. | Bronchitis. | Faucitis. |

* I regard faucitis (inflammation of the fauces, or epidemic sore throat) and diphtherite as the same disease; but have confined the name diphtherite to the cases where a deposit of lymph took place.—D. M.

| | | |
|---|---|---|
| 11. | Diphtherite, bronchitis, faucitis, | Faucitis, uterine hæmorrhage. |
| 12. | [colic. | Faucitis, dyspepsia. |
| 14. | Colic, faucitis. | Faucitis, conjunctivitis, catarrh. |
| 15. | Faucitis, diarrhœa, bronchitis. | Faucitis, bronchitis. |
| 16. | Faucitis. | Faucitis, vertigo. |
| 17. | | Faucitis, hæmicrania. |
| 19. | Faucitis. | Frostbite, dysentery. |
| 20. | Faucitis, diarrhœa, dysentery. | Diarrhœa. |
| 21. | Diphtherite. | Faucitis. |
| 22. | Diphtherite, faucitis. | Dysentery. |
| 23. | Amenorrhœa- pleurodynia. | |
| 24. | Faucitis, diarrhœa, abortion. | Periostitis, diarrhœa, lumbago. |
| 25. | Diarrhœa,with cramps & vomiting. | Purulent ophthalmia. |
| 26. | Faucitis, diarrhœa, dysentery. | Diarrhœa, herpes, fever, conjunctivitis, |
| 27. | Catarrh, bronchitis. | Gravel. [epilepsy. |
| 28. | Faucitis, diarrhœa. | |
| 29. | | Faucitis, dysentery, menorrhagia. |
| 30. | Faucitis, choleraic diarrhœa. | |
| 31. | Faucitis, choleraic diarrhœa, | Hæmorrhoids. |

1858. January.

| | | |
|---|---|---|
| 1. | Bronchitis. | |
| 2. | Faucitis. | Bronchitis, diarrhœa. |
| 3. | Faucitis. | Bronchitis. |
| 4. | Faucitis, bronchitis, dysentery. | Bronchitis. |
| 5. | Faucitis, bronchitis. | |
| 6. | Phthisis, convulsions. | |
| 7. | Faucitis. | Bronchitis, colic. |
| 8. | Faucitis, bronchitis, dysuria, | Faucitis with diarr., dropsy, lumbago. |
| 9. | [thrush. | Porrigo scutulata, frostbite, herpes. |
| 10. | Typhoid, congestion of brain, bronchitis. | Synovitis. |
| 11. | Faucitis, hooping cough. | Bronchitis, congestion of brain,eczema |
| 12. | Faucitis, abortion, bronchitis, hooping cough, diarrhœa. | Vertigo, struma. |
| 13. | Rheumatism, bronchitis, gastro- | Catarrh, rheumatism, bronchitis. |
| 14. | Gastrodynia. [dynia, dysentery. | Catarrh, bronchitis. |
| 15. | Diphtherite, dysentery, pleuro- | |
| 16. | Scarlatina. [dynia. | |
| 17. | Faucitis, pleurodynia, diarrhœa, bronchitis. | Epilepsy. |
| 18. | Faucitis, with diar. & bronchitis. | Bronchitis, diarrhœa. |
| 19. | Faucitis, dysentery. | |
| 20. | | Bronchitis. |
| 21. | Faucitis. | Diarrhœa, catarrh, hooping cough. |
| 22. | Faucitis with mumps, abortion. | Prostatitis, dyspepsia. |
| 23. | Diarrhœa. | Bronchitis. |
| 24. | Diarrhœa, epilepsy. | Faucitis. |
| 25. | Bronchitis, hæmorrhage. | Prurigo. |
| 26. | Catarrh, hysterical epilepsy. | Dysentery, porrigo. |
| 27. | Bronchitis, diarrhœa, tonsillitis. | Bronchitis. |
| 28. | Diarrhœa. | Diarrhœa, catarrh. |
| 29. | Bronchitis. | Diarrhœa, bronchitis, catarrh, tænia. |
| 30. | Faucitis with dysentery, hooping cough, diarrhœa, incipient paralysis, emphysema. | Otorrhœa, faucitis, diarrhœa. |

February.

| | | |
|---|---|---|
| 1. | Hysteria, hooping cough. | Diarrhœa, catarrh, bronchitis, chil- |
| 2. | Faucitis, diarrhœa. | Catarrh. [blain, insanity, |
| 3. | Bronchitis, hooping cough. | Bronchitis. |
| 5. | Faucitis with dysentery, catarrh. | Diarrhœa. |

| | | |
|---|---|---|
| 7. | Faucitis with bronchitis, small-pox, bronchitis. | Colic. |
| 7. | Diphtherite, scarlatina. | Frostbite. |
| 8. | Faucitis, bronchitis, gout, dysentery, diarrhœa. | |
| 9. | Scarlatina, menorrhagia. | Erythema, dyspepsia. |
| 10. | Faucitis, bronchitis, stomatitis ulcerosa, pleurodynia. | Bronchitis. |
| 11. | Faucitis with mumps. | Faucitis, adenitis. |
| 12. | | Faucitis. |
| 13. | Diphtherite, scarlatina. | Faucitis, catarrh, diarrhœa, scabies. |
| 14. | Dysentery. | Faucitis, pleurodynia. |
| 15. | Faucitis, bronchitis, catarrh, dysentery. | Pneumonia. |
| 16. | Diarrhœa, catarrh. | |
| 17. | Diphtherite, faucitis, hooping cough, bronchitis. | Dyspepsia. |
| 18. | Bronchitis, hooping cough, congestion of brain. | Diarrhœa, bronchitis. |
| 19. | Epistaxis, tetanus. | |
| 20. | Epilepsy. | Colic, adenitis. |
| 21. | Bronchitis, pleurisy, syncope. | |
| 22. | Faucitis with eczema rubra, syncope. | Faucitis, bronchitis, phthisis, dyspepsia. |
| 23. | Bronchitis, dysentery. | |
| 24. | Faucitis, bronchitis, tonsillitis, fever, hooping cough. | Rheumatism. |
| 25. | Epilepsy. | Bronchitis. |
| 26. | Pleurodynia, stomatatis ulcerosa, tonsillitis. | Anasarca. |
| 27. | Faucitis, urticaria. | Conjunctivitis. |
| 28. | Faucitis, parotitis. | |

"The diarrhœa, dysenteria, and bronchitis, were chiefly among children. 67 paupers were attended, as fresh cases, in December; 64 in January: and 54 in February. Of these, 22 were between 60 and 70 years old; 19 between 70 and 80 years old; 12 between 80 and 90 years old; and one, who had been living in poverty and wretchedness for many years, died of bronchitis at the great age of 94.

The subjoined meteorological report is furnished by Mr. Dyson:—

"There has been very little variety in the weather to record during the quarter ending February 28th. The temperature was mild in the first two months and the greater part of the last; it reached 57° on the 22nd December; 56° on the 3rd January; and 51° on the 5th February; but fell, during the first night of the same month, to 19°, or 13° below the freezing point. The barometric pressure was generally uniform, and for the most part above 30 inches. The sky was usually clear, and the atmosphere seldom disturbed by any storm, till the first week in March. The wind blew chiefly from the south and south-west till February, and during that month cold north-easters prevailed. The amount of rain which fell in December was ·21 inches; in January ·44 inches; and in February ·42, making a total of very little more than one inch of rain in three months. Ozone was freely developed from the 2nd to the 8th, and from the 16th to the 22nd of December; in January, from the 4th to the 10th, and from the 24th to the 31st. In February, from the 2nd to the 9th, and from the 17th to the end of the month. 'Everybody' has remarked 'what a mild winter we have had.'"

*Liverpool.*—"During the quarter (including 14 weeks) ending the 2nd January, 3849 deaths were registered in Liverpool, including 136 on which inquests had been held. The number is 230 more than the corrected average of the same period of former years. That this excess was chiefly owing to atmospheric causes, is shown by the fact, that 1258 deaths were caused by diseases of the lungs, while the corrected average is only 1028, the mortality from these diseases having been thus more than 22 per cent. above the average. Zymotic diseases caused 1125 deaths, about 9 per cent. more than the average. Of these, scarlatina was the most fatal, having caused 397 deaths. Typhus caused 175, being more than in any previous corresponding period since the Irish epidemic of 1847-48. Hooping cough caused 200 deaths, diarrhœa 136, small-pox 59, (more than in any quarter of the previous three years), croup 27, measles 23, syphilis 18."

For the deaths in each week and the meteorological returns, see the table on next page.

*Hindley, Wigan.*—Mr. Hussey writes: "There is nothing particular to record this quarter. The wind for the most part has been easterly. In my own district, I never saw less sickness."

*York.*—Mr. Procter says: "There has been a considerable amount of general indisposition during the quarter. Small pox has been very general, but on the decline in February. There has also been a considerable amount of scarlatina, but mild in its form, and not fatal in the majority of cases. Diphtherite has frequently shown itself, with gastric irritation; but I have neither met with, nor heard of, any cases of death from this cause."

*Lerwick.*—Dr. Spence writes: "December, January, and the first half of February, were extremely changeable, boisterous, wet, and stormy, there being few days on which rain did not fall, and few nights on which it did not blow a violent gale. The prevailing wind was south-west. There were two falls of snow, one in January, and the other in February, but it did not lie for more than twenty-four hours. The latter half of February was remarkably fine, and unusually dry; so much so, that there was some scarcity of water. The prevailing winds were easterly. Comparing this with the corre-

### MORTALITY AND METEOROLOGY OF LIVERPOOL.

| DEATHS. Week ending | December. | | | | January. | | | | | February. | | | |
|---|---|---|---|---|---|---|---|---|---|---|---|---|---|
| | 5th. | 12th. | 19th. | 26th. | 2nd. | 9th. | 16th. | 23rd. | 30th. | 6th. | 13th. | 20th. | 27th. |
| Scarlet fever* | 28 | 23 | 34 | 20 | 28 | 17 | 24 | 16 | 24 | 26 | 22 | 26 | 18 |
| Measles | 3 | 1 | 4 | 2 | 3 | 3 | 11 | 7 | 13 | 14 | 26 | 17 | 12 |
| Small-pox | 3 | 4 | 12 | 4 | 5 | 8 | 4 | 9 | 8 | 14 | 9 | 12 | 10 |
| Hooping cough | 16 | 8 | 12 | 15 | 9 | 11 | 10 | 9 | 15 | 11 | 9 | 8 | 11 |
| Croup | .. | .. | 3 | 2 | 2 | 2 | 1 | .. | 5 | 4 | 7 | 4 | .. |
| Chicken-pox | .. | 2 | .. | .. | .. | .. | 1 | .. | .. | .. | 1 | .. | .. |
| Cholera | .. | .. | .. | .. | .. | .. | 1 | .. | .. | .. | .. | .. | .. |
| Diarrhœa | 6 | 6 | .. | 11 | 3 | 3 | .. | 6 | 4 | 4 | 9 | 1 | 3 |
| Typhus | 11 | 8 | 16 | 12 | 2 | 15 | 11 | 14 | 6 | 12 | 11 | 10 | 8 |
| Diphtheria | .. | .. | .. | .. | .. | .. | .. | .. | .. | 2 | 1 | 3 | .. |
| Diseases of lungs | 127 | 114 | .. | 78 | 82 | 94 | 90 | 87 | 94 | .. | 93 | 18 | 87 |
| Scurvy† | .. | .. | .. | 1 | .. | .. | .. | .. | .. | 1 | 1 | .. | .. |
| Intemperance | 1 | 1 | 2 | 2 | .. | .. | 1 | 1 | .. | 3 | .. | .. | .. |
| Syphilis | 2 | 1 | 1 | 3 | 1 | 2 | .. | .. | 3 | .. | .. | 2 | 1 |
| Total deaths during each week | 297 | 257 | 268 | 247 | 205 | 259 | 258 | 263 | 268 | 276 | 309 | 275 | 255 |
| **METEOROLOGICAL OBSERVATIONS.** | | | | | | | | | | | | | |
| Barometer: Mean | 29·95 | 30·41 | 30·10 | 30·13 | 30·47 | .. | 30·33 | 30·43 | 30·13 | 29·79 | 30·12 | 30·07 | 30·08 |
| Dew-point temperature | 42·8 | 43·8 | 42·3 | 43·7 | 39·9 | .. | 41·2 | 36·0 | 35·4 | 35·3 | 30·4 | 32·6 | 26·4 |
| Thermometer: Highest (mean) | 50·0 | 53·4 | 51·1 | 53·0 | 48·1 | .. | 49·8 | 45·8 | 45·5 | 46·4 | 42·0 | 42·5 | 44·3 |
| Lowest (mean) | 41·6 | 44·6 | 44·6 | 47·1 | 42·2 | .. | 42·4 | 39·7 | 38·0 | 37·1 | 33·6 | 33·2 | 32·4 |
| Mean | 45·3 | 49·0 | 47·9 | 50·1 | 45·2 | .. | 46·1 | 42·8 | 41·8 | 41·8 | 37·8 | 37·9 | 38·3 |
| Mean of 11 years | 41·3 | 43 5 | 43·3 | 40·5 | 40·6 | .. | 39·7 | 40·9 | 40·3 | 41·2 | 41·3 | 41·6 | 42·4 |
| Range | 8·4 | 8·9 | 6·5 | 5·7 | 5·9 | .. | 7.5 | 6·1 | 7·5 | 9·3 | 8·4 | 9·2 | 11·9 |
| Mean range, 11 years | 6·8 | 6·8 | 6·8 | 5·9 | 5·8 | .. | 5 8 | 6·0 | 6·4 | 6·6 | 6·7 | 7·3 | 7·4 |
| Rain fell on days | 2 | 2 | 3 | 3 | 2 | .. | 3 | 2 | 1 | 1 | 1 | 1 | 0 |
| Rain fell during hours | 5 | 1·7 | 12·5 | 8 | 1·2 | .. | 2·7 | 1·7 | 5·7 | 0·8 | 1·5 | 1·2 | 0 |
| Amount of rain in inches | 0·21 | 0·03 | 0·73 | 0·24 | 0·03 | .. | 0·14 | 0·11 | 0·16 | 0·05 | 0·05 | 0·03 | 0 |
| General direction of wind | SSE. | S. | S. W. | W. | SW. SSE | .. | W. | W. N. | SSE. | var. | SE. | E. | SE. |

* Zymotic diseases have lately been much more prevalent in Liverpool than for some months previously, and the mortality generally has been greater.
† The cases of scurvy occurred in sailors, who came from ships as they arrived from distant voyages, and were entirely cases imported into Liverpool.

sponding quarter of last year, we find the difference to consist in the absence of small-pox and fever, and the prevalence of dysentery. Diarrhœa was more common, and erysipelas less so than last year, while the prevalence of catarrh and influenza was about the same in both years. I saw several cases of aphthous ulceration of the mouth; but though on the outlook for them, I met with no cases of diphtherite. A few mild cases of scarlet fever occurred in an adjoining parish; they could not be traced to any source. The total number of deaths during the quarter was 19: the average age was 37, there being five below 5, and eight above 70. The causes, in those cases in which medical certificates were furnished, were: bronchitis, 2; phthisis, 2; infantile remittent fever, 2; apoplexy, 2; epilepsy, 1; erysipelas, 1; chronic hepatitis, 1; and debility from old age, 3."

